# Food allergy: cause or consequence of pediatric eosinophilic esophagitis? Potential implications of ultraprocessed foods in prevention and management

**DOI:** 10.3389/falgy.2023.1138400

**Published:** 2023-06-29

**Authors:** Laura Carucci, Martina Votto, Amelia Licari, Gian Luigi Marseglia, Roberto Berni Canani

**Affiliations:** ^1^ Department of Translational Medical Science, University of Naples “Federico II,” Naples, Italy; ^2^ ImmunoNutritionLab at the CEINGE Advanced Biotechnologies Research Center, University of Naples “Federico II,” Naples, Italy; ^3^ Pediatric Unit, Department of Clinical, Surgical, Diagnostic and Pediatric Sciences, University of Pavia, Pavia, Italy; ^4^ Pediatric Clinic, Fondazione IRCCS Policlinico San Matteo, Pavia, Italy; ^5^ European Laboratory for the Investigation of Food-Induced Diseases, University of Naples Federico II, Naples, Italy; ^6^ Task Force for Microbiome Studies, University of Naples Federico II, Naples, Italy

**Keywords:** Th2 inflammation, esophageal barrier, advanced glycation end products, alarmins, ultraprocessed foods

## Abstract

Eosinophilic esophagitis (EoE) is a chronic, immune-mediated disease characterized by eosinophilic infiltration, leading to esophageal dysfunction, inflammation, and fibrotic remodeling. In the last few decades, there has been an increased prevalence of EoE at an alarming rate in the pediatric age. The pathogenesis of EoE is still largely undefined, and this limits the definition of effective strategies for the prevention and management of this condition. EoE is considered a multifactorial disease arising from a negative interaction between environmental factors and genetic background, causing an impaired esophageal epithelial barrier with subsequent abnormal allergen exposure activating type 2 (Th2) inflammation. Food antigens have been suggested as key players in Th2 inflammation in pediatric patients with EoE, but emerging evidence suggests a potential role of other dietary factors, including ultraprocessed foods, as possible triggers for the occurrence of EoE. In this paper, we discuss the potential role of these dietary factors in the development of the disease, and we propose a new approach for the management of pediatric patients with EoE.

## Introduction

Food allergy (FA) in children is a major health concern, with an increased prevalence in the past two decades (1–4). Different clinical phenotypes of FA have been described, all deriving from the alteration of the mechanisms of immune tolerance to dietary antigens (5). Concomitantly, a similar increase in the prevalence of eosinophilic esophagitis (EoE) has been observed in the pediatric age (6–8). Children affected by FA present an increased risk of developing EoE later in life, and now EoE is considered as a component of the allergic march (9). EoE is a chronic disease characterized by an eosinophilic inflammation of the esophagus and symptoms of esophageal dysfunction (10, 11). Like FA, EoE is considered a condition deriving from a negative interaction between genetic background and environmental factors, leading to esophageal barrier dysfunction. The esophageal barrier alteration facilitates an abnormal exposure to dietary antigens and the consequent activation of type 2 (Th2) inflammatory response (6, 12). EoE has evolved from a rare condition to a commonly encountered disease in pediatric clinical practice and a significant cause of upper gastrointestinal morbidity (13). The global prevalence of EoE is 0.5–1 cases/1,000 persons (13). In children, the pooled incidence of EoE is 6.6 cases/100,000 person years, whereas the pooled prevalence is 34 cases/100,000 children (14). During the last few years, several studies reported a dramatic increase in EoE prevalence, especially in children in Western Countries (7, 14–16). Although this evidence might be related to improved medical awareness and knowledge, it could also be related to the global increase in allergic disorders. Despite some genetic factors have been associated with an increased risk of developing EoE, environmental factors seem to be the most relevant players facilitating the occurrence of the disease (13). In the last few years, one of the most impressive changes in the exposure to environmental factors concerns dietary habits. The consumption of ultraprocessed foods (UPFs) rapidly spread in the last few decades among children living in Westernized countries (17, 18). Increased exposure to UPFs is considered a facilitating factor for the occurrence of several chronic non-communicable diseases, including FA (19, 20).

In this paper, we discuss the potential role of UPFs and FA in the development of the disease, and we propose a new approach for the management of pediatric patients with EoE.

## Genetic and environmental factors: an intriguing interplay in the pathogenesis of EoE

The pathogenesis of EoE is still largely undefined. It is commonly considered a multifactorial disease in which genetic and environmental factors may play a role. These factors, through intricate and bidirectional interactions, are responsible for esophageal barrier impairment, with loss of cell-to-cell adhesion mechanisms (desmosomes, tight, and adherence junctions), increased permeability, and consequent abnormal exposure to dietary antigens (21, 22). Alteration of the esophageal barrier leads to the epithelial release of inflammatory molecules such as thymic stromal lymphopoietin (TSLP) and interleukin (IL)-33, also called alarmins. These mediators drive the differentiation of Th2 effector cells, with the consequent production of several Th2 cytokines (IL-4, IL-5, IL-9, and IL-13) and massive recruitment of eosinophils (23). Simultaneously, luminal antigens encountering antigen-presenting cells (APC), activate specific antigen Th2 differentiation, induce additional release of inflammatory cytokines, eosinophils recruitment, and plasma cell activation with specific IgE production (23).

### Lessons from genetic findings

The role of genetic factors in EoE pathogenesis was postulated with the observation that disease prevalence varies among sex and ethnicity. Epidemiological studies show that EoE is most common in white males, in children, and in adults (24–26). Genetic susceptibility is also supported by the evidence that having a first-degree family member affected by EoE increases the risk for disease occurrence (OR, 16.3; 95% CI, 9.4–28.3) (27). The relevance of the genetic background has also been supported by the results of candidate-gene and genome-wide association studies (GWAS), highlighting the role of different *loci* involved in the Th2 inflammatory response, and in the regulation of epithelial barrier structure and function in patients with EoE (12, 28, 29). The integrity of the esophageal epithelial barrier is ensured by desmosomes, tight and adherence junctions, as well as by several genes involved in epithelial cell differentiation, including filaggrin (FLG) and desmoglein 1 (DSG1). A genetic variation in these genes was detected in patients with EoE (24). The most powerful association has been found in the alteration of calpain 14 (CAPN14) production, an enzyme involved in esophageal barrier regulation via the IL-13 pathway (30, 31). Lastly, two other variations in serine peptidase inhibitors, kazal type 5 and 7 (SPINK5 and SPINK7), were also detected in barrier integrity maintenance (12, 28, 29).

EoE is characterized by a Th2 inflammatory response and a high prevalence of other atopic comorbidities (32, 33). Several genetic alterations were detected in patients with EoE, mainly related to the Th2 response, resulting in the upregulation (up to 53-fold) of eotaxin-3 (CCL26), TGF-β, and Periostin (POSTN), respectively, involved in eosinophil chemotaxis and adhesion, with the consequent production of TSLP (12, 34). TSLP is considered a crucial mediator involved in the EoE inflammatory cascade. Although TSLP is also expressed in other atopic disorders, TSLP production seems unrelated to other concomitant allergic diseases in patients with EoE (28, 29).

Despite this evidence, twin studies reporting a low disease concordance in both monozygotic (41%) and dizygotic (22%) twins suggest the greater importance of environmental factors as a major driving force for the occurrence of EoE in genetically predisposed children (35, 36).

### The potential role of environmental factors in facilitating the occurrence of EoE

Growing evidence underlines that early life exposure to several detrimental factors, as already reported for FA pathogenesis, could promote esophageal barrier dysfunction and Th2 inflammatory response in EoE (37–39). In contrast, several beneficial environmental factors, such as breastfeeding and the Mediterranean diet, showed a protective role against these conditions (40–42).

Several environmental agents could induce esophageal barrier dysfunction. This could be the case of the detergents that altering the epithelial barrier, induced mucosal inflammation and the typical histological features of EoE in a preclinical model (43). Immortalized esophageal epithelial cells (EPC2) exposed to sodium dodecyl sulfate (SDS), a widely used detergent contained in domestic cleaning, cosmetic, pharmaceutical, and food products, showed a significant decrease in transepithelial electrical resistance and a significant increase of FITC-dextran flux. In addition, a proinflammatory IL-33 mRNA expression and a reduction of DSG1 expression were detected, with consequent alteration of epithelial barrier integrity. It was also observed that mice exposed to SDS showed a marked activation of proinflammatory cytokine pathways and esophageal eosinophilia compared with not-exposed controls (43).

Like FA, infections have also been proposed as potential risk factors for the occurrence of EoE. Some case series showed a direct association between herpes simplex virus esophageal infection and the development of EoE (44).

Data regarding different living areas are discordant. Some studies showed a positive association between EoE occurrence and suburban areas (36, 45, 46). It is well known that rural vs. urban or suburban areas are characterized by a considerable difference in pollution exposure, aeroallergen content, and climate temperature, which can modify the allergen air concentration. Living in a cold climate zone seems related to a higher risk of EoE occurrence, but more studies are needed to support this hypothesis (47). Aeroallergens have long been proposed as a trigger or worsening factor for EoE (48, 49), but their role in the pathogenesis of EoE is still controversial. Indeed, if it is well known that aeroallergens induce a Th2-orientated immune response in other allergic diseases (i.e., allergic rhinitis or asthma), their role in EoE occurrence or exacerbation needs to be better investigated (50–53). Recent studies on the role of seasonality were unable to demonstrate significant differences in EoE occurrence and disease course (54, 55).

New studies are now exploring the potential role of the Western diet as a trigger for non-communicable disease occurrence, including FA (19, 20, 56). Western diet is low in fibers and polyunsaturated fats and rich in UPFs (57). During the last few decades, the consumption of UPFs significantly increased in children living in Western countries. It was estimated that 65% of the total daily energy intake derives from UPF consumption in children in the US and EU (18, 19).

Smith and colleagues highlighted how dietary patterns could be related to FA occurrence in children (20, 56). They linked different types of foods consumed by US children and fast-food consumption by Australian pediatric subjects, with the increase in FA prevalence (58, 59).

Furthermore, countries with a huge increase in the EoE, FA, and anaphylaxis rates were also the countries where Western diet rapidly spread among the child population in the same period (7, 14–16, 60–65).

One of the main UPF-derived compounds are the advanced glycation end-products (AGEs), deriving from the non-enzymatic reaction between proteins and sugars via the Maillard reaction (66).

Dietary AGEs activate several inflammatory pathways, including the Th2 inflammatory response, through interaction with specific receptor (RAGE) expressed by epithelial cells, peripheral blood mononuclear cells, human esophageal mucosal cells, and by human eosinophils (17, 67–69). The activation of RAGE induces several intracellular pathways that activate the alarmins signal with increased production of TSLP, IL-33, and IL-25 (70). These inflammatory cytokines exert a pivotal role in EoE and FA pathogenesis, and they induce differentiation of innate lymphoid cells 2 in Th2 effector cells with a consequent production of IL-4, IL-5, IL-9, and IL-13 (12). AGEs also activate mast cells, via RAGE activation, with a consequent release of proinflammatory cytokines, and may induce the production of specific IgE against dietary antigens (71, 72). In addition, dietary AGEs increase oxidative stress levels, and may also act at the gastrointestinal (GI) level by impairing gut microbiome structure and function and tight junction protein expression (56, 73). These proteins are crucial in maintaining the esophageal and gut barrier integrity; thus, an increased epithelial permeability allows an abnormal antigen passage (56, 74). In summary, the alteration of the gut and esophageal barrier integrity, the abnormal antigen translocation, and the alarmin activation with a consequent Th2-orientated response, may allow an altered antigen presentation, resulting in a potentially harmful condition for the maintenance of immune tolerance to dietary antigens (75, 76).

Lastly, it has been demonstrated that proton pump inhibitors (PPIs) could modulate both esophageal barrier integrity and alarmin signal (77). This could be an additional mechanism of action of PPIs in EoE treatment.

Altogether, these data, from the epidemiological and immunological points of view, add plausibility to the potential role of UPFs in facilitating the occurrence of EoE and FA.

### Food allergy-EoE links

As EoE is characterized by Th2 inflammatory response, most pediatric patients have other coexisting atopic comorbidities, such as FA, allergic oculorhinitis, and asthma (32, 33). This clinical picture demands multidisciplinary management involving pediatric allergy, gastroenterology, and nutrition expertise (78). Observational studies have demonstrated that the risk of developing EoE increases in allergic children, especially in those with ≥1 allergic disease, and to date, EoE has been proposed as a component of the allergic march (9). Moreover, allergic sensitization has been reported in most pediatric patients with EoE (79). According to recent FA classification, EoE can also be considered a mixed (IgE- and non-IgE-mediated) FA, where food antigens have been proposed as triggers for esophageal Th2 inflammation in genetically susceptible patients (80). In 2017, a systematic review with the meta-analysis by Gonzalez-Cervera et al. reported that the frequency of FA in patients with EoE, compared with healthy controls, ranged from 0% to 44%, with a relevant clinical heterogeneity in FA definition (81). Thereafter, Capucilli and Hill, assessing the prevalence of allergic diseases in patients with EoE, reported a 24%–68% prevalence from 2015 to 2019 (33). A more recent literature revision confirmed that the prevalence of IgE-mediated FA varies between 25% and 70% (31).

The *primum movens* in allergic diseases is the epithelial barrier alteration, as in the case of FA (21). After this, the loss of immune tolerance against allergens is crucial for FA development (33). In the context of IgE-mediated disease, specific IgG4 are generally increased and considered a marker of immune tolerance. Evidence shows that patients with EoE may also present high levels of IgG4, but their role in EoE pathogenesis and diagnosis is unclear. In fact, EoE shares some clinical features not only with IgE-mediated FA but also with IgG4-related disease, characterized by progressive fibrosis (82). In 2014, Clayton et al. showed increased IgG4-positive plasma cells (IgG4-PC) in the lamina propria and granular extracellular IgG4 deposits in adults with EoE. In addition, the authors reported high IgG4 serum levels against milk, wheat, egg, and nuts in these patients, demonstrating that the esophageal deposition of IgG4 was associated with food-specific IgG4 antibodies (83). Recent studies confirmed the presence of total specific IgG4 high serum level in pediatric patients with EoE compared with healthy controls (84, 85). Unfortunately, despite this evidence, the pathogenetic role of IgG4 in EoE is still unclear and requires further research.

FA and EoE have also been linked by the response to the elimination diet (32, 86–88). However, despite the fact that a complete clinical response to the elimination diet is observed in all children with FA, as this is mandatory for making a definite diagnosis of FA (89), the response to the elimination diet has not been reported in all children with EoE (90). The first evidence that foods were the triggers of esophageal inflammation were reported by Kelly et al. (91). The authors highlighted the link between FA and EoE by showing that children treated with an exclusive elemental (amino acid–based) formula completely recovered from GI symptoms and showed a drastic decrease in esophageal eosinophilia (91). The elemental diet is effective in up to 90% of pediatric patients with EoE (90).

The most frequently implied foods in pediatric patients with EoE are cow's milk, wheat, soy and/or legumes, egg, tree nuts, and shellfish. The elimination of these food allergens showed different efficacy rates, depending on the number of foods removed and the rationale used to eliminate them from the diet (empirical vs. targeted) (92, 93). The empirical elimination of all these six food antigens produced effective results in approximately 72% of patients, and the targeted one could induce a similar remission rate in patients with EoE when a combination allergy screening tests is performed [skin-prick tests (SPT), atopy patch tests (APT), and/or specific IgE] (90, 94). The four-food elimination diet (cow's milk, wheat, soy, and egg) induces histological remission in above 53% of patients, with higher efficacy in children than in adults (60% vs. 46%) (90). Kagalwalla et al. performed a prospective observational study in children with EoE treated with a four (cow's milk, wheat, egg, and soy)-food elimination diet finding that after food reintroduction, the most common food triggers that induced histologic inflammation were cow's milk (85%), egg (35%), wheat (33%), and soy (19%) (93). Therefore, since milk and wheat are the most allergenic foods, Molina-Infante et al. proposed starting with an empirical 2-food elimination diet, finding that this approach was effective in 43% of treated patients (95). The authors thus proposed a step-up approach that avoids unnecessary dietary restrictions and spare GI endoscopies to assess histologic remission (96). A recent prospective study in children with EoE found that the single milk elimination diet was effective in more than 50% of patients, suggesting that this dietary intervention may be proposed as first-line treatment because of the ease of implementation and adherence (96). More recently, de Rooij et al. proposed a mixed dietary treatment in adults with active EoE, combining the empirical four-food elimination diet with an amino acid–based formula. The authors found that, although the combined dietary treatment significantly improved the quality of life in adult patients with EoE, it did not lead to a more considerable decrease in the peak of eosinophil count at 6-week follow-up (97). As already reported in patients with atopic dermatitis, children with EoE may develop IgE-mediated hypersensitivity to food antigens (98, 99). On the other hand, children who outgrow IgE-mediated FA and reintroduce the culprit food(s) in their diet, can later develop EoE for the same food (100).

Unfortunately, the response to the food-elimination diet is not complete or sustained over time in many children with EoE (90). Several factors impacting clinical or histologic response should be considered in patients with EoE who are unresponsive to the elimination diet (Table 1) (101).

**Table 1:** Established and possible causes of unresponsiveness to food elimination diets and suggested solutions.

Causes of unresponsiveness	Solutions
**Low diet compliance**	
Poor palatability of amino acid-based formula Several dietary restrictions Expensive cost of amino acid-based formula or dietary alternatives Psychosocial isolation with negative impact on the quality of life Desire to consume trigger foods	Discuss with patient and his/her family all possible therapeutic strategies
	Modified elemental diet (amino acid-based formula + one or two less allergenic foods, generally vegetables or fruits)
	Nasogastric tube or gastric tube in candidate children*
	Nutritional and psychological counseling
**Food contamination**	Patient and family education
**Persistent fibro-stenotic disease with esophageal stricture**	Esophageal dilatation
**Persistent high exposure to other environmental/ dietary factors (**ultraprocessed foods, detergents**)**	Patient and family education Nutritional counseling

* Toddlers and young children with active disease complicated by severe failure to thrive and malnutrition.

## Discussion

In the last few decades, the increased incidence and prevalence of pediatric EoE paralleled with the increased incidence, prevalence, and severity of the clinical manifestations of FA, in the pediatric age. The origin of these parallel epidemiologic patterns is still largely undefined, but it could be the target for innovative preventive and therapeutic strategies against both conditions.

The role of dietary factors in EoE pathogenesis has been long considered only from the FA point of view, in which food antigens are considered triggers for the esophageal barrier dysfunction, for the occurrence of Th2 inflammatory response and the consequent clinical and histological features of EoE (10). It is now time to speculate that the abnormal food antigen exposure could be just the consequence of a first hit, which could be mainly responsible for the occurrence of EoE in genetically predisposed individuals (21). Thus, defining which environmental factor could elicit the first hit could be paramount for designing disrupting strategies against EoE.

The activation of alarmins is one of the initial signals in EoE pathogenesis, driving a Th2 inflammatory response and esophageal barrier alteration (79, 102). Recent data suggest that selected environmental factors could induce alarmins signal and esophageal barrier dysfunction (20, 103). Among these factors, the UPF detrimental compounds, AGEs, seem to be relevant candidates able to directly “switch on” EoE inflammation (20). AGEs directly activate the production of alarmins. Then, esophageal barrier impairment could be responsible for increased epithelial permeability and abnormal exposure to food allergens, with subsequent sensitization of food antigens (24). This could explain why sensitization of food antigens is commonly observed in pediatric patients with EoE. In the light of this, sensitization of FA and food antigens cannot be the trigger but just an epiphenomenon in several pediatric patients with EoE. This could justify why the response to the food-elimination diet may be ineffective in a number of children with EoE.

Altogether, it is possible to hypothesize that UPFs, and in particular dietary AGEs, could act as the *primum movens* for the esophageal barrier dysfunction, mimicking the innate alarmin pathways and facilitating the occurrence of EoE in genetically predisposed children. This hypothesis could drive innovative preventive measures to limit UPFs/AGEs exposure in the pediatric age and provide a new strategy for EoE management. This could be a reasonable, affordable, and easily applicable strategy against EoE.

**Figure 1:**
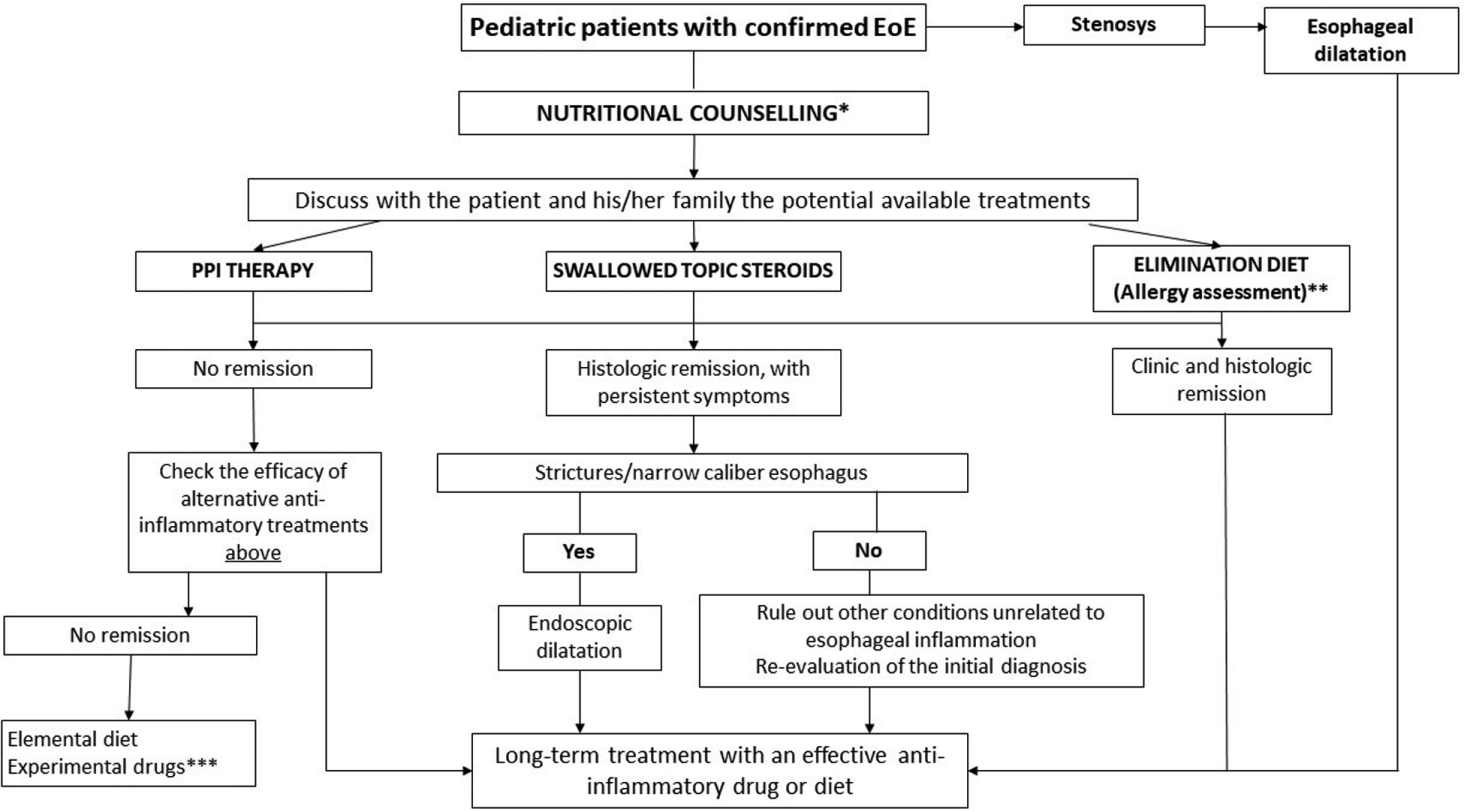
Toward an innovative strategy for the management of pediatric EoE. EoE, eosinophilic esophagitis; PPI, proton pump inhibitors. *Nutritional counseling aims to assess the patient's diet, eating habits, potential nutritional deficiencies, and reduces exposure to ultraprocessed foods. **Skin prick test, specific IgE, and atopy patch test + nutritional counseling. ***Consider biological treatment (dupilumab was approved by the FDA in patients >12 years with EoE).

Thus, we propose a new approach for pediatric EoE management, in which nutritional counseling aimed to reduce exposure to UPFs/AGEs could facilitate better therapeutic outcomes in pediatric patients with EoE (Figure 1). Future preclinical and clinical studies are advocated to explore the potential of this approach.
